# The Physiology of Diet and Hygiene at the North Pole

**Published:** 1923-01

**Authors:** Cecil Clarke


### Critical Review.

### THE PHYSIOLOGY OF DIET AND HYGIENE AT THE NORTH POLE.

Tales of polar adventure interest most men from the mere recital of human endeavours and of hardships endured, but they have a special appeal to the medical mind. It is sometimes a relief to escape from the atmosphere of infectious disease, and apart from gastro-intestinal disorders, illness is rare in these latitudes. The Scott expedition, for example, enjoyed freedom from such complaints until the opening of a bale of clothing, when an epidemic of coughs and colds occurred.

The points of special interest to the medical reader are those relating to dietary, personal hygiene, and housing.

Vilhjálmur Stefánsson, the leader of the Canadian Arctic expedition (1914 to 1918), would appear to have solved these problems for the northern regions. In his book[1] this explorer records the work accomplished during a period of five years, and at the same time seems to destroy deliberately and with intent many of our illusions as to the dangers, discomforts, and hardships met with in this charmed circle. Some acquaintance with the literature of Antarctic travel inclines one to the belief that the latter continent will remain still, in the classic phrase, deliberately unfriendly.

[1] *The Friendly Arctic*, by Vilhjálmur Stefánsson.

As a preliminary to understanding the problem of life at the North Pole, Stefánsson advises us to forget what we think we already know. "Unless it be religion, there is no field of human thought where sentiment and prejudice take the place of sound knowledge and logical thinking so completely as in dietetics." Thus Stefánsson, who enunciates the principle that the essence of dietetics in the North is to choose a complete food, *e.g.* fat caribou or seal meat, and to avoid variety in the diet. We can well believe that he had great difficulty in persuading his fellow-travellers to act up to these theories. Peary, with a standard diet, remarks that his men ceased to grumble after the second week. Stefánsson found his men liked the unchanged diet better the longer they were confined to it, and he states that it is easier to persuade men "well brought up," *i.e.* accustomed to a varied liberal diet, than men "poorly brought up," *i.e.* used to only half a dozen or so articles, to carry out what was really a dietetic experiment. He was, in fact, adopting the ideas of the inhabitants of these parts, the Eskimo and the polar bear. Now the physiologist states we must have so much protein, fat, carbohydrates, salt, and water of 2,500 calories or so value. The polar bear obtains his requirements from seal blubber almost exclusively, and moreover avoids in this way all the evils of excess of protein. All practical experience supports the contention that there is a great need for fat in the Arctic diet. The caloric value of fat is more than double that of any other food, and to obtain the equivalent of 1 lb. of fat 2¼ lb. of sugar is required—no small addition to the weight of rations. Stefánsson remarks that four centuries ago, before sugar was known in Europe, the phrase to "live on the fat of the land" had some significance. However, it has none now; we live on the carbohydrates of other lands. What is not generally appreciated is that there are degrees of flavour in fat, palatability depending on the site of origin. Thus the *bonne-bouche* in caribou is the retro-ocular and patellar fat; this applies to unseasoned, underdone boiled fat. Marrow is softer and more palatable the nearer it is to the extremity of a limb.

The standard polar ration of previous expeditions allows very little variety:—

*Peary's:* Pemmican, 1 lb., ship's biscuits 1 lb., condensed milk 4 oz., compressed tea ½ oz. Total, 36½ oz.

*Scott's:* Pemmican 12 oz., biscuit 1 lb., sugar 3 oz., butter 2 oz., compressed tea 7 oz., cocoa 0.6 oz. Total, 34.3 oz.

Pemmican is a prepared and condensed food consisting of beef, fat, and dried fruits. Its most essential constituent is fat. Scott's supply contained 60 per cent., and was found most satisfactory. Stefánsson considers it should contain at least

50 per cent. fat. Some of his pemmican, deficient in this respect, caused serious ill-health to the men and actual starvation to those dogs living solely on pemmican. Viewed from the standpoint that the diet was too rich in protein, the author states that " protein poisoning " occurred. " This leads to nephritis or derangement of the kidneys, of which a common symptom is swelling of the body, beginning usually at the ankles." Whilst fully cognisant of the fact that excessive protein intake causes renal irritation (the eating of 2 lb. of beef-steak by healthy men has been followed by microscopical hæmaturia), a more likely explanation of the swelling is *hunger œdema*. Briefly stated, this condition is due to a combination of semi-starvation and hard muscular work ; the essential lack in the diet is either fat or special vitamine, such as fat-soluble A. In the case under consideration the two factors, hard work with severe exposure, and deficient fat in the food, were present for at least five months. Indian tribes in Canada and Hudson's Bay men occasionally have to live mainly on protein (lean meat) ; they refer to it as starvation.

Stefánsson finds that the standard polar diet produces thirst, and although this can be easily quenched by snow, he considers it is preferable not to become thirsty. But his chief objection is that a prepared food, such as pemmican, requires to be transported many miles from civilisation, and that it takes up valuable sledge room in the Arctic zone. The greatest service rendered by him to future North Polar expeditions is the development and proof of his theory that it is possible always to live " off the land " even if afloat on an ice floe in the Arctic Ocean, providing always that the explorer is an experienced hunter, capable of immense physical efforts and learned in the science of polecraft. If on the sea ice, the food supply is mainly seal and to a less extent polar bear ; if on land, there are abundant caribou deer and many polar oxen. The broth in which the meat selected is cooked provides the desirable hot fluid.

Three cases of scurvy resulted from a diet of " groceries." Definite symptoms were noticed at the end of three months. Stefánsson gives them as follows : failure of strength but not of appetite as an early sign ; irritability (" showing itself in a tendency to condemnatory and uncalled-for argumentativeness ") ; dizziness on standing ; disinclination for exertion ; and depression. It would, one imagines, under the conditions of life in the Arctic, be quite possible to have some of the above symptoms apart from scurvy, but the well-known physical signs of swollen and loose and aching teeth would help to differentiate the two conditions. Slightly cooked or raw fresh caribou meat and marrow effected a quick cure in about three weeks. In the Scott expedition seal blood was used by Dr. Atkinson for a similar purpose.

It has often occasioned comment that a scurvy patient hankers for salt, and that this longing disappears as the cure proceeds; but no explanation can be given. Although it has long been recognised that salt meats predispose to scurvy, the relationship of salt to the disease is considered to be only indirect. Many Indians and Eskimos find salt unpalatable and do not use it at all. The rôle of salt in the civilised world is so universal that few have ever been in the position to appreciate its absence, as were some of the members of Stefánsson's expedition. The greatest longing was felt after some two or three weeks' deprivation; but after being without it for some time, six to eight weeks, the men did not want it. Salt has once furnished the material for an entry in an explorer's diary. Bowers' of Scott's expedition, wrote on January 25th, 1912: "I am in tribulation as regards meals now, as we have run out of salt, one of my favourite commodities." He goes on to say that he "will only have to *endure* another two weeks without it."

Peary (from experience) would have no one who smoked or required alcohol. The objections to tobacco were that it had a bad effect on the "wind," that it was an additional and useless weight to carry, that it vitiated the atmosphere of the igloo, and, doubtless the soundest reason of all, that the smoker is a nuisance to himself and others when his supply gives out.

The thought of starvation has been robbed of some of its terrors since hunger-striking became so effective a reply to the majesty of the law. And it is a familiar fact that the preliminary hunger caused by missing the first meals does not become more but slightly less compelling as time elapses. Stefánsson states that after continued strenuous exertion—walking 20 to 30 hours—without food, he became no more hungry than if he had missed a meal by a few hours—a tribute to his physical fitness, and an experiment suitable for confirmation by some keen student. Like everyone else, if in camp he could eat every four hours or so, and it annoyed him to find that his men could not be persuaded that a man does not collapse from hunger if he fails to get a snack every few hours. He found, to his surprise, that the greatest irregularity had neither the effect of diminishing nor of increasing hunger. In the North one gets hungry sooner if one gets cold: this seems no unusual experience.

Hitherto explorers confronted by a shortage of rations have kept, in Stefánsson's opinion, the sensation of hunger tantalisingly acute but never at bay by going on half rations or less. He himself, under similar conditions, would continue on full rations and trust to the North to provide his food. This philosophy would be of no use to the explorer in the South.

A belief that has handicapped polar explorers in the past is the idea that if a man goes to sleep when lost he never wakes, the cold being supposed to induce sleep. This is contrary to our everyday experience that it is impossible to get to sleep if we are cold; as a matter of fact, if we become cold when asleep we wake up. The keeping awake at all costs resolves itself, he considers, into walking quickly up and down in semi-panic; perspiration makes the clothing a good conductor of heat; until at last further loss of heat and exhaustion usher in the final sleep. Stefánsson, when in the open, found he could not sleep more than a quarter of an hour without being woken because of feeling chilled.

One of the alleged hardships of life at the poles is the long spell of darkness which has to be endured (110 days). Captain Evans writes, "It was difficult to imagine that in three months there would be no sun, and that the cold dark winter would be upon us." This from an R.N. Officer on his fourth Antarctic trip! But to the Eskimo it is the social period; during the winter he visits his friends and relations, and his wife alone works. In fact, one Eskimo privately regarded this dread of the darkness as one of the peculiarities of white men which he did not understand. The feeling that darkness is to be dreaded is apparently a question of temperament: the Manchurian ponies of Scott's expedition cheered up greatly with the return of the sun, the officers of the expedition drank champagne, and Bowers composed poetry. Peary was the first to realise that the late winter is the best time for a sledge journey; March and April, with intense cold and perpetual light, are the best months.

It is a mistake to consider the Arctic lifeless or silent. There are meadows and grass-eating animals (polar oxen), abundant flowering plants, and mosquitos to plague the life of man and beast. Noise is supplied by the crunching of the ice-floes of the polar pack. On the Antarctic continent all writers note the absence of life and even noise in that "sad, frozen land." Amundsen, on his return journey from the South Pole, records apparently with pleasure the fact of seeing a skua gull, significant of the near neighbourhood of the coast. However, so far inland as 200 miles from the Pole, Evans records without comment that a skua gull was seen.

Even at the North Pole the housing question affords, most appropriately, material for heated discussions. Stefánsson condemns the double silk tent except for summer and for temporary use. For him the snowhouse or igloo, built as by the Eskimo. Two important modifications, however, are made by him ; first, the door is on a level with the floor, or a little below it, and in accordance with the principle that hot air, being lighter than cold, rises, the cold air in the doorway remains there, and, it is stated, no draught results ; secondly, a hole is made in the roof for ventilation, its size depending on the requirements of the inmates (*i.e.* whether sleeping or cooking) and on the conditions of external temperature. It is from the Eskimo-inhabited igloo that the classical specimens of solid atmosphere come ; his test for the need of more oxygen is either the lamp going out, or a colleague developing the convulsions of asphyxia. Yet he can smell the smoke of a single dwelling when eight miles away ! Such snow houses were built by the explorer's party just within an hour, and it is hardly credible that the kerosene or seal oil stove easily maintains a warmth within the house of 60° to 80° F. While Europe and America were on fuel rations in the winter of 1917–18, his men were sitting in shirt sleeves in snow houses built on the floating polar ice, with unlimited fuel, *i.e.* seal-blubber.

Stefánsson devised satisfactory clothing and footwear, and also solved the problem of keeping hoar-frost out of the inner clothing. That this is an achievement of the very greatest importance to all future explorers may be gathered from the following figures. At the start of their heart-breaking trip to Cape Crozier, the three sleeping bags of Wilson, Bowers, and Cherry-Garrard weighed 471 lb. ; on arrival home the weight was 118 lb. Evans, in his *South with Scott* writes most feelingly of the clammy misery his party endured. The final upshot is, that when the hoar-frost in the clothing and the sleeping bag has thawed out, the unfortunate occupant spends the night in what is practically an ice-water bath. The only occasions on which Stefánsson was unable to avoid getting wet and cold were when travelling through melting snow *in the summer !*

It may be asked : Did the Canadian party become aware of the lack of anything ? Of two things only : the first, the chance of hearing good music ; the second, alarm clocks. The manner in which Browning, of Lieutenant Campbell's party in the Scott expedition, overcame the latter deficiency deserves to be known. A candle burning through a thread rove through it releases a bamboo spring, which, attached to the trigger of a gramophone, starts a tune which is dinned into the ear of the required sleeper, who turns out and stops the tune. This contrivance could be timed to five minutes.

It is probably no exaggeration to say that one or more volumes exist on every polar expedition made. Yet none of them seems to comment on a matter which must be of great significance in such extremes of temperature, *i.e.* advice as to the technique of the everyday calls of nature when on the march or in camp. As the extreme example of inclemency, let us visualise Cape Crozier with a wind on one occasion of 84 miles per hour and a temperature of 77° below zero. To the uninitiated the performance of such a requirement might attain the dignity of a polar hardship.

Despite Stefánsson's modest disclaimers, it is very clear that his great achievements are due entirely to his knowledge and ability to meet all the hard conditions of life in these zones. His lieutenant, Storkerson, also a man skilled in polecraft, was able to write as follows of one of his journeys: "We lived on sea ice uninterruptedly for 238 days, having experienced neither hunger nor thirst, danger nor hardship," *i.e.* a journey of over 200 miles on moving and treacherous ice in darkness, fog, and storm. Further, this party of 5 men and 16 dogs, leaving with 101 days' full rations, kept themselves for 8 months (96 seals and 6 polar bears were procured).

As an example of Stefánsson's personal ability, consider for a moment his method of finding camp when in difficulties. The account that follows is a much condensed description. He was in a region where only three kinds of wind blow, from S.W., from N.E., and from E.N.E. An examination of the ground will easily show which wind blew last. By observing the size and characteristics of drift, he could tell at a glance which was the S.W., which the N.E., and which the E.N.E. And if it were dark or a blizzard were blowing, he could tell the shapes of the drifts by stopping and feeling with the feet or on all fours *with the hands*. Then having determined either the N.E. or the S.W. drifts, the only problem is to cross every such drift at the required angle and you arrive at your camp. Could anything be simpler! After a performance of this kind, when he had been on his feet about 17 hours, he says: "I was, of course, not tired." He was, as he truly remarks, in good training. And he had solved an interesting problem (how not to get lost in a blizzard), he was in especially good humour, so the time passed happily.

CECIL CLARKE.

